# The Physicians Prescription Book

**Published:** 1871-10

**Authors:** 


### Books Reviewed.

*The Physicians Prescription Book;* containing lists of the Terms, Phrases, Contractions, and Abreviations used in Prescriptions. By Jonathan Pereira, M. D. Lindsay & Blakiston, Philadelphia.

This little book is designed for the use of the Medical and Pharmaceutical Students. It is divided into three parts. The first consists of general remarks on prescriptions; the second part is upon prescriptions in an abreviated form; the third part unabreviated prescriptions with literal translations The book has been revised by the author and has reached its fifteenth edition. It contains much valuable information upon all subjects connected with Pharmacy, and will prove itself valuable to both Physicians and Pharmacist.

